# Developing the perceived social support scale for older adults: A mixed-method study

**DOI:** 10.3934/publichealth.2020007

**Published:** 2020-02-06

**Authors:** Shima Nazari, Pouya Farokhnezhad Afshar, Leila Sadeghmoghadam, Alireza Namazi Shabestari, Akram Farhadi

**Affiliations:** 1 Department of Gerontological Nursing, School of Nursing and Midwifery, Tehran University of Medical Sciences, Tehran, Iran; 2 Department of Gerontology, School of Behavioral Sciences and Mental Health (Tehran Institute of Psychiatry), Iran University of Medical Sciences, Tehran, Iran; 3 Department of Nursing, School of Nursing, Social Development and Health Promotion Center, Gonabad University of Medical Sciences, Gonabad, Iran; 4 Department of Geriatric Medicine, School of Medicine, Tehran University of Medical Sciences, Tehran, Iran; 5 Department of Nursing, School of Nursing and Midwifery, Bushehr University of Medical Sciences, Bushehr, Iran

**Keywords:** elderly, perceived social support, scale development, validation

## Abstract

Social support has an important impact on the well-being of the elderly. Some studies have shown that perceived social support is more important than received social support. Perceived social support has different definitions across different age groups and cultures. So, this sequential exploratory mixed-method study was designed to develop and validate a perceived social support scale for community-dwelling elderly. In the qualitative phase, the perspectives of the elderly on perceived social support were defined through directed content analysis. Then, an extensive item pool was designed based on the elderly's perception and review of the literature. In the quantitative phase, the validity (content, face, and construct) and reliability (internal consistency, stability) of the newly developed scale was assessed using the sampling of five hundred elderly. The final scale consists of 34 items with domains of “emotional support”, “practical support”, “spiritual support”, “negative interactions” and “satisfaction with support received” that explained 58% of the total variance of the scale. The internal consistency varied from Cronbach's α = 0.70 to 0.87 for the subscales and as 0.92 for the whole scale. The study showed that the scale as a valid and reliable instrument can be used for the proper measurement of perceived social support among the elderly.

## 1. Introduction

Many studies have demonstrated that social support is one of the most influential components of social determinants of health, has a positive impact on the physical and mental health of elderly [1]–[3].

Social support is the social network that a person receives help when needed (including family, friends, and neighbors) [4]. It is stated that the elderly's perceptions of the availability of social support are thought to be more important than received support [2]. Perceived Social Support (PSS) is a multifaceted and complex concept for which there is no agreed single definition. In general, this functional component of social support refers to the perceived adequacy of a variety of types of support (emotional, practical, informational and financial) and an individual's satisfaction with support exchanges [5],[6]. The great impact of PSS on the health and well-being of the elderly is for the design of appropriate interventions to promote it. However, to evaluate, plan and implement these interventions, we need to accurately measure this concept.

Some scales were originally designed for the assessment of PSS in adults, which are commonly used in studies with elderly, although these were neither specifically designed for elderly, nor were they designed from the perspective of this particular age group. However, it seems as though the appropriate and holistic study of PSS demands that researchers consider the contribution of the target group themselves, in addition to the researcher-made theories [7]. An overview of these instruments revealed that most of them did not assess all different aspects of PSS comprehensively, especially in the elderly [8],[9].

Numerous studies have shown that PSS is hugely dependent on social context and cultural norms [10]. People from different cultural backgrounds but with similar support networks tend to be differently influenced by social support and to have different expectations of support and its perceived benefits [11]. Neglecting to acknowledge such diversity can complicate the appropriate assessment of support interventions [12]. For instance, the elderly's traditional perception of support is typically shaped by the pivotal role played by family bonds and the duty for caring for seniors in extended family households [13]. Indeed, the seniors preferred the support provided by their close people especially their children rather than formal support. However, the lack of sufficient studies on the domain of the elderly's perception of support with scales designed based on cultural norms and social context has been highlighted. Most studies on PSS have been conducted in western countries with individualistic culture, which may not be suitable for resolving the same problem in countries with a collectivist culture [14]. Hence, it is necessary to assess PSS under the current social conditions in each country. So, this study aimed to develop a scale that would specifically measure PSS in community-dwelling elderly and to evaluate its psychometric qualities.

## 2. Materials and methods

The sequential Exploratory mixed study was designed according to classic measurement theory in several stages [15] including the directed content analysis on semi-structured interviews, the review of literature, the construction of the extensive item pool; assessing its content and face validity; performing a field study, psychometric evaluation and statistical analysis of the newly developed scale.

### 2.1. Qualitative phase

Iranian older adult's perspectives on PSS were investigated using directed Content Analysis based on Elo and Kyngas (2008) works. This deductive method is reexamined to present data about the concept in a new context by developing a constrained matrix based on theories, models, literature reviews, and coding interview texts according to these pre-designed categories. Codes outside this matrix can be used in producing new categories [16].

The matrix was designed using a comprehensive review of previous studies conducted on perceived social support, which containing categories of emotional, practical, informational, social companionship, providing supports, conflicts, and satisfaction with these supports.

#### 2.1.1. Participants

Community-dwelling elderly living in different zones of Tehran participated in this study. Inclusion criteria were those community-dwelling elderly, aged 60 and older, normal cognitive abilities (AMT ≥ 7) and the ability and willingness to take part in the study.

#### 2.1.2. Interviews

In an individual, semi-structured interviews, the open-ended general questions were asked such as “Describe, your communication with others during last week, please” to encourage participants to express emotions and perceptions, and continued with exploratory questions such as “what kind of help do you think you can count on? Who?” Further questions were asked according to the participant's answers and interview guidelines. The interviews continued until no new codes were revealed and the saturation of data fulfilled [17]. The audio recordings of interviews reviewed according to the matrix and converted into meaning units and codes. Various codes were compared in terms of differences and similarities in the presence of three researchers, and the codes consistent with matrix categories were grouped in those categories, and new codes grouped to make new categories. To further increase rigor and trustworthiness of data, we utilized extended fieldwork and confirmed the results through member checks and audit trials.

#### 2.1.3. Item pool

The PSS concept and its subcategories were defined from the elderly's viewpoints and taken as the core for constructing the item pool. The relevant literature and the available PSS instruments were searched and their items, which did not exist in the above definition, were included in the primary pool. To reduce the risk of social desirability bias and respondent measurement error, several items were worded negatively [18],[19].

### 2.2. Quantitative phase

In this phase, the psychometrics evaluation was conducted from July to October 2017. The size of the sampling was different depending on each stage of work (same inclusion criteria). Socio-demographic data were examined using age range, gender, marital status, education level, living condition and source of income.

#### 2.2.1. Content validity

Qualitative content validity was determined through a panel of experts, including scale developers, gerontologists, geriatrics and sociologists, examining the scale in terms of its wording, grammar, appropriate phrasing and correct placement of the items. Then the quantitative content validity, including content validity ratio (CVR), item-content validity index (I-CVI) and scale-content validity index-average (S-CVI-Ave), of the tool were assessed [20],[21]. The CVR was used for assessing the necessity of the items using Lawshe's method [22]. We calculated I-CVI and S-CVI-Ave to assess the relevance of the items along with the computing a multi-rater kappa coefficient [21].

#### 2.2.2. Face validity

The qualitative face validity of the scale was confirmed by asking a convenience sample of thirty community-dwelling elderly to evaluate the apparent fitness of the scale's name and items, their ability to understand the text, its readability and level of difficulty and whether they felt comfortable responding to the items [23].

The quantitative face validity of the scale was measured using the item impact score, and each item >1.5 was considered to be acceptable [24]. The elderly assessed the importance of each item based on a 5-point Likert scale, ranging from “5 = It is totally important” to “1 = It is not important at all” [25].

#### 2.2.3. Field study

A field study was conducted using a convenience sample of community-dwelling elderly aiming to determine the initial quality of the scale and to reduce the number of its items [18],[26].

#### 2.2.4. Construct validity

To assess the construct validity of the designed scale, exploratory factor analysis (EFA) and convergent validity relationship between the PSS scale and the 12-item General Health Questionnaire (GHQ-12) were evaluated.

The EFA was performed using the “Principal Component Analysis” method with “Varimax rotation”. Before the EFA, the Kaiser-Meyer-Olkin Sampling Index (KMO) (sufficiency of sample size) and Bartlett's Test of Sphericity (appropriateness of correlation matrix) were calculated [18]. A minimum factor loading of 0.4, eigenvalues ≥ 1.5 and the scree plot was used to determine the number of factors, which retained [27],[28].

Previous studies have shown that PSS is linked to mental health [29]. Therefore, the correlation between subcategories of GHQ-12 and PSS scale was tested on a sample of elderly (as a part of a sample of EFA). GHQ-12 is a self-report measure of psychological morbidity in community settings which comprises of six “positively phrased items” expressing positive descriptions of mood states and six “negatively phrased items” expressing negative descriptions of mood states which were scored using a 4-point Likert scale ranging from 0 to 3 [30]. The psychometrics study showed that the Iranian version of the GHQ-12 is a reliable and valid instrument that can be used for measuring psychological health in Iran (Cronbach's α = 0.87) [31]. (Current study Cronbach's α = 0.92).

#### 2.2.5. Reliability

Cronbach's α (internal consistency) for the whole scale and its subscales and Test-retest (stability), along with interclass correlation coefficient (ICC) were used for evaluating the reliability of the scale [32]. A Cronbach's alpha and ICC of 0.70 was set as the lowest acceptable measure, and scores between 0.80 and 0.90 were generally assumed to be very good [27],[33]. The elderly selected through convenience sampling completed the scale twice, with a two-week interval and under the exact conditions. The ICC between the scores obtained from the two tests was then determined. The data were analyzed using SPSS-22.

### 2.3. Ethical considerations

The participants were briefed on the research, including the aim of the study, that their participation was voluntary, the confidentiality of the data, and their right to withdraw from the study at any time without any consequences. Written informed consent was obtained from each participant.

## 3. Results

### 3.1. Qualitative phase

Eleven elderly women and seven elderly men aged between 65 years and 86 years (mean 72.8 years in women and 76.8 years in men) participated in interviews. Of them 8 were widowed, 9 were married, and one had never married. Participants' education levels varied from high school to university degree. Among women, 3 had retired and the rest were housewives. All men were retired.

A total of 968 initial codes were extracted from the analysis of data. Elimination of repetitive codes and integration of similar ones finally led to 83 codes in 8 main categories, including emotional, practical, informational, social companionship, providing, spiritual (new category) support, conflicts and satisfaction with support (Table 1) [34].

The experiences and perspectives of the Iranian elderly on perceived social support were revealed.

The initial item pool consisted of 98 items (86 positive and 12 negatives), according to these categories and a review of the literature was designed. The items were scored based on a 5-point Likert scale, from “5 = totally agree” to “1 = totally disagree”, and scores were reversed for the negative items.

**Table 1.** Dimensions Of elderly PSS based on qualitative analysis.

Category	Subcategory
Emotional support	Interaction with closed people
	Love and affection
	Caring
	Confidant
	Affectionate security in need
	Positive self-perception
Practical support	Tangible helps
Informational support	Appraisal
	Problem solving
Social companionship	Social integration
Providing support	Providing nurturance
	Being responsible to others
Spiritual support	Trust
	Spiritual coping
Conflicts	Negative interactions with others
	Dependency to others
	Social neglect
	Psychological pressures
Satisfaction with support	Satisfaction of others
	Satisfaction of life

### 3.2. Quantitative phase

Based on the twelve experts' opinions, three items were merged and 33 items were edited concerning the writing style.

A total of 41 items with a CVR of less than 0.75 were eliminated. All I-CVI and S-CVI-Ave values were above 0.78 and 0.8 respectively, which considered appropriate when there were nine or more experts [35],[36]. The kappa coefficient was found to be favorable (above 0.76) for all the I-CVI values.

According to thirty elderly's opinions, the 5-point Likert scale, from “5 = totally agree” to “1 = totally disagree”, was converted to “5 = very much” to “1 = very little” (qualitative face validity). The impact score exceeded 1.5 for all the items and no item was therefore eliminated for failing to meet this criterion.

In the field study, the 55-item scale was firstly completed through the interview with fifty community-dwelling elderly and the total Cronbach's α calculated, which was 0.47. Then, after reviewing the scale, seven items were removed as they had a value > 0.5 in the Cronbach's α “if item deleted” column in SPSS. As a result, the total of Cronbach's α was improved to a value of 0.71 after deleting seven items. At the end of this stage, a 48-item scale was prepared for the psychometric evaluation stage.

Five-hundred eligible elderly (at least 10 samples for each item) were randomly selected through stratified cluster sampling from various zones of Tehran (north, south, center, east, west) including 55.2% women (n = 276) and 44.8% men (n = 224). They ranged from 60–90 years of age, and consisted of 68% of people aged 60–75 years, and 32% aged 76–90 years. Most of them were married (63%), illiterate (54.4%), lived with their spouse and children (42.6%), and were retired (55.4%) (Table 2).

**Table 2.** Socio-demographic characteristics of the community dwelling elderly.

N = 500	N	%
Gender		
Male	224	44.8
Female	276	55.2
Age		
60–75	340	68
76–90	160	32
Marital status		
Married	315	63
Divorced	31	6.2
Widowed	136	27.2
Never married	18	3.6
Education		
Illiterate	272	54.4
Reading & writing	131	26.2
High school	75	15
University degree	22	4.4
Living arrangement		
Spouse	102	20.4
Spouse & Children	213	42.6
Children	104	20.8
Other family	8	1.6
Alone	73	14.6
Source of income		
Retirement	277	55.4
Pensioner	105	21
Dependent if children's support	27	5.4
Other includes personal assets and no income	91	18.2

The authors verified that a sufficient number of elderly of various socioeconomic statuses were included (maximum variation). The elderly were asked to rate the scale based on their experiences of social support over the past six months. A KMO index of 0.92 and Bartlett's score of 7994.143 was considered statistically significant at p < 0.0001, highlighting that distinct factors could be extracted from the factor analysis. The findings showed that 58% of the total variance of the scale could be explained by the first five factors; 18.797, 10.123, 8.305, 7.796 and 7.362, respectively (Table 3).

**Table 3.** Exploratory factor analysis of older adult´s perceived social support scale.

1. Affective support (Eigenvalue = 9.023, accounted for 18.797 of variance, Cronbach's α = 0.87)	Factor loading
Q3 Presence of confidence	0.60
Q4 Close people's willingness to have elderly among them in their gatherings	0.52
Q6 Being adored	0.53
Q7 Being useful	0.71
Q8 Being approved	0.63
Q10 Unburdening themselves to close people	0.77
Q11 Having close people's sympathies	0.62
Q12 Receiving love and affection	0.76
Q16 Being deemed important	0.62
Q21 Receiving care and attention	0.65
Q23 Being respected	0.70
Q26 Being trusted	0.64
Q27 Having intimate relationships	0.80
Q29 Enjoying the company of close people	0.66
Q31 Being supported	0.79
Q33 Encourage close people to pursuit of interests by elderly	0.58
2. Practical support (Eigenvalue = 4.859, accounted for 10.123 of variance, Cronbach's α = 0.78)	
Q1 Interaction with close people	0.61
Q2 Borrowing money	0.80
Q17 The availability of close people for help	0.54
Q22 The availability of the older adult for help	0.68
Q24 Care during times of illness	0.61
Q25 Spending leisure time	0.58
Q28 Help in daily routine tasks	0.61
3. Spiritual support (Eigenvalue = 3.986, accounted for 8.305 of variance, Cronbach's α = 0.79)	
Q5 Resorting to religious practices	0.79
Q13 Participation in acts of charity	0.69
Q14 Being prayed for by close people	0.59
Q18 Being hopeful	0.66
Q30 Having God's support	0.72
4. Satisfaction of received support (Eigenvalue = 3.742, accounted for 7.796 of variance, Cronbach's α = 0.74)	
Q19 Being on the receiving end of appreciation from close people	0.67
Q20 Showing appreciation to close people	0.56
Q34 Satisfaction with the interaction with close people	0.77
5. Negative interactions (Eigenvalue= 3.534, accounted for 7.362 of variance, Cronbach's α = 0.70)	
Q9 Being pressured by excessive expectations	0.62
Q15 Limited freedom of action	0.62
Q32 Feeling lonely	0.62

**Figure 1.**
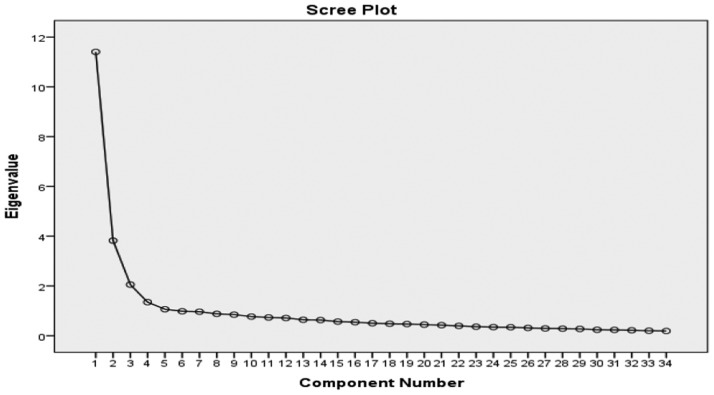
Scree plot of 34 Item exploratory factor analysis.

The scree plot also showed a steep decline in eigenvalues after the fifth factor (Figure 1). Consequently, after eliminating 21 items in the EFA and confirmation of the five-factor structure of the scale, the number of scale items was reduced to 34 items in five subscales. Each factor was named according to the content of its constituent items, which were: “emotional support” (16 items), “Practical support” (7 items), “Satisfaction with received support” (3 items), “Spiritual support” (5 items), and “Negative interactions” (3 items). The subscales within the scale correlated positively from moderately to high levels with one another, varying from 0.30 to 0.78 (Table 4).

**Table 4.** Pearson Correlation matrix between subscales of older adult´s perceived social support scale.

Variable	Affective Support	Practical Support	Spiritual Support	Satisfaction of received support	Negative Interactions
Affective Support	1				
Instrumental Support	0.582 *	1			
Spiritual Support	0.514 *	0.425 *	1		
Satisfaction of received support	0.781 *	0.592 *	0.421 *	1	
Negative Interactions	0.551 *	0.583 *	0.301 *	0.603 *	1

* Correlation is significant at the 0.01 level (2-tailed).

There was a moderate to high (r = 0.39–0.79, P < 0.01) correlation between domains of emotional, practical, spiritual support, the satisfaction of received support, and “positively phrased items” of GHQ-12. There was also a correlation between negative interaction and “positively phrased items” of GHQ-12 (r = −0.67, P < 0.01) (Table 5).

After the removal of the poorly fitting items, the Cronbach's α value was calculated as 0.70–0.87 for the subscales, and as 0.92 for the whole scale on a sample of thirty elderly. The ICC was 0.84–0.98 (P < 0.01) for the subscales and 0.91 for the whole scale (assumed fit).

**Table 5.** Final variables associated with mental health level in the multiple linear regression models.

Variable	Unstandardized Coefficients		Standardized Coefficients β	t	Sig.
	B	Std. Error			
(Constant)	42.337	1.417	-	29.872	<0.001
Affective Support	−0.300	0.050	−0.441	−6.066	0.000
Practical support	−0.065	0.075	−0.044	−0.871	0.385
Spiritual support	−0.006	0.072	0.003	0.085	0.932
Satisfaction of received support	−0.612	0.167	−0.235	−3.666	<0.001
Negative interaction	0.609	0.101	−0.270	−6.010	<0.001

Adjusted R2 = 0.814.

## 4. Discussion

The present study was conducted to develop and test the psychometric properties of an appropriate scale for the assessment of PSS in community-dwelling elderly. This scale includes not only the positive aspects of support in the elderly but also negative interactions, as well as the mutual support provided between the elderly and those with whom they have a close relationship.

The final 34-item PSS scale (31 positive and 3 negative) suits the population of the elderly and has been designed according to their particular perspectives of support (qualitative content analysis). To lighten the burden of responding to the scale for the elderly, various types of support were assessed without considering the source of support (family, friends and neighbors) [37]. This scale can be easily used in diverse settings and takes only 20 minutes to complete. To preserve the integrity of its psychometric properties, the scale must be completed through an interview, even in the case of literate elderly. Also, the overall scale and each subscale can be scored separately.

Unlike the results of the content analysis (eight dimensions), we found five subscales in the EFA. These results emphasize the multidimensionality and complexity of the PSS construct, which has a close relationship with social and cultural contexts. Therefore, the different studies revealed different attributes for measuring PSS [2],[38],[39]. For example, among the most used general PSS scales, the brief Duke-UNC Functional Social Support Questionnaire (DUFSS) measured PSS just by assessing emotional support from an intimate relationship. The Lubben Social Network Scale assessed the size, closeness, and frequency of interactions between the elderly and their families, friends, and neighbors [9]. The Multidimensional Scale of Perceived Social Support (MSPSS) measured emotional/informational, tangible, emotional support, positive social interaction and number of friends and neighbors. However, our scale consisted of emotional, practical, spiritual support, satisfaction with received support and negative interactions.

The first subscale was labeled “emotional support”, based on the nature of the items involved. The expectation of the availability of emotional support influences mental health significantly [40]–[42]. Therefore, emotional assistance is considered to be the most significant type of support for helping an individual to cope with a variety of stressors [2],[43]. The importance of this factor justifies its status as the dominant factor of the new scale and that it contains the majority of the items (16 items). Emotional support will be more emotional when the elderly perceive that they could also provide it to those that they are close to [14]. Therefore, in this new scale, the subscale, “encouraging close people to pursue interests enjoyed by elderly”, which was categorized as “providing support” in the item generation phase, was moved into the emotional support subscale. The special structure of Iranian families means that older parents prefer to stay closely involved with their children's issues throughout their lives, including into adulthood. For example, most children live with their parents until they are married. Therefore, it is not surprising that the elderly feel a duty to encourage their children to follow their interests and consider such encouragement to be a form of emotional assistance.

The second subscale was named “Practical support”. Physical changes due to aging tend to generate a need for instrumental assistance from those with whom they have a close relationship [44],[45]. In countries such as Iran, this role becomes more important as there is a serious lack of efficient formal support systems.

The third subscale was labeled “Satisfaction with received support”. Being satisfied with a relationship, as a key concept of PSS [46], can be considered the best indicator of subjective well-being in the elderly. Other studies have shown that the Iranian elderly's satisfaction with life and spirit has a significant relationship with social support, but they did not assess the satisfaction of support itself.

The fourth subscale was named “Spiritual support”, which has not generally been considered in other common PSS scales. The mosque plays a remarkable role in the beliefs, attitudes and daily lives of Iranians. Many religious ceremonies in the Islamic culture of Iran bring people together. Previous studies have shown that some elderly turn to religion and spirituality to cope with their life stressors and problems [47],[48]. However, few studies have addressed spiritual support as a dimension of social support and the majority have referred to spirituality as a separate concept called “Spiritual health” [49], which helps to improve individuals' social support. The position of religious beliefs within the Iranian culture is a special so, this factor was considered as a part of the elderly' PSS.

Finally, the fifth subscale was called “Negative interactions”. The assessment of the elderly' PSS should also include the potential costs incurred because the adverse effects of negative interactions with those they are close to can exceed the overall benefits of interactions [50],[51]. Because of the Iranian elderly' reliance on their close relationships and the transitional nature of recent changes in cultural, social, and economic circumstances, it is essential to evaluate conflicts and negative interactions in addition to the positive aspects of PSS.

Three categories were extracted from the directed content analysis, including “Informational support”, “Companionship support” and “Providing support”; however, in the factor analysis, the “Companionship” items were divided into “Practical” (spending leisure time), “Affective” (enjoying the company of close people) and “Spiritual” support (participation in acts of charity). Because of the serious lack of formal social support infrastructures in Iran, the poor public transport systems and the elderly's expectations of spending much time with family members, they have to rely on close people for recreation and traveling as instrumental assistance. Alternatively, spending more time with their close family members than others (neighbors, friends, and others) made the Iranian elderly feel emotionally satisfied [52]. Considering the religious background of the Iranian elderly, they feel good when allocating time and energy to charitable activities. They enjoy attending mosques and religious centers to build up their spiritual storage for what they believe to be life after death [34],[53].

The “Informational” items comprised guidance; receiving advice and information from close people were eliminated. A high percentage of the Iranian elderly (more than 63%) are illiterate [54],[55], therefore, they are not able to read books or newspapers or to use new technologies such as the internet. As a result, they are limited to obtaining the most necessary information from TV. At the same time, transitional changes in socio-cultural status lead to reduced opportunities for the exchange of ideas and views between the elderly and their close ones, because young people tend not to have enough time or interest (they perceive that elderly' knowledge is outdated and unusable (in being with them [53]. This shift in the cultural situation reveals the need for further studies on this subject.

The relationship between subcategories of the PSS scale and GHQ-12 demonstrates that a high level of PSS leads to less anxiety and depression symptoms and high levels of social function and self-esteem [56],[57]. Therefore, we can conclude that a high level of PSS could increase the quality of life and well-being of the elderly.

### 4.1. Strengths and limitations

As the first scale designed for assessing PSS in elderly in Iran, this modified PSS scale was specifically developed to target elderly by obtaining their perceptions to contribute to the meaning of social support, combined with a review of the literature and multiple stages of evaluation by conducting a precise course of validity and reliability methods. Hence, this instrument is a unique means of obtaining a favorable view of PSS for the elderly, particularly in the presence of drastic changes in the social and cultural conditions in low and middle-income countries, and given the increasing aging population. Another strength of this study is that the psychometric evaluation was completed with a sufficiently large sample of elderly (n = 500) with various socioeconomic statuses (drawn from the various zones of Tehran.)

One limitation of the study is the completion of the scale through the interview. Variation in the interviewers' skills and experience with the elderly may have influenced the participants' responses. Therefore, interviewers need adequate training to be more familiar with the process of scale completion. Another weakness is that the Cronbach's-α (internal consistency) for one subscale (negative interactions) is less than 0.7, but this is probably due to its low number of items (three items).

## 5. Conclusion

The PSS scale is a valid and reliable brief instrument, which gives an overall score of elderly' PSS. It can be used by gerontologists and other healthcare professionals for research purposes, for screening elderly who are at risk of low social support, developing emotional interventions for promoting it, and, last not the least in evaluating outcomes of interventions. Moreover, social policymakers will be able to consider the different aspects of PSS in developing specific support programs for the elderly, particularly in countries such as Iran, which have recently experienced the most rapid changes in traditional social structures. It appears that this revised PSS scale can better evaluate elderly' PSS in societies with similar socio-cultural conditions, especially by emphasizing concepts such as negative interactions, satisfaction with the support received, and spiritual support. We suggest conducting more studies in different socio-cultural contexts to further confirm the psychometric properties of the scale and increasing its generalizability.
