# Supplementary material for: Developing the perceived social support scale for older adults: A mixed-method study
Source: AIMS Public Health. 2020 Feb 6;7(1):66–80. doi: 10.3934/publichealth.2020007 (PMC7109536; doi:10.3934/publichealth.2020007)
Supplement: Supplementary file 1 [file publichealth-07-01-007-s001.pdf]

AIMS Public Health

http://www.aimspress.com/journal/aimsph

AIMS Public Health, 7(1): 66–80.
DOI: 10.3934/publichealth.2020007
Received: 25 November 2019
Accepted: 23 January 2020
Published: 06 February 2020

Research article

Developing the perceived social support scale for older adults: A mixed-method study

Shima Nazari[1], Pouya Farokhnezhad Afshar[2,]*, Leila Sadeghmoghadam[3], Alireza Namazi Shabestari[4] and Akram Farhadi[5]

[1] Department of Gerontological Nursing, School of Nursing and Midwifery, Tehran University of Medical Sciences, Tehran, Iran
[2] Department of Gerontology, School of Behavioral Sciences and Mental Health (Tehran Institute of Psychiatry), Iran University of Medical Sciences, Tehran, Iran
[3] Department of Nursing, School of Nursing, Social Development and Health Promotion Center, Gonabad University of Medical Sciences, Gonabad, Iran
[4] Department of Geriatric Medicine, School of Medicine, Tehran University of Medical Sciences, Tehran, Iran
[5] Department of Nursing, School of Nursing and Midwifery, Bushehr University of Medical Sciences, Bushehr, Iran

* **Correspondence**: Email: farokhnezhad.p@iums.ac.ir; Tel: +982163471352.

Appendix

Table S1. The Perceived Social Support Scale.

	Items	Very high	High	Low	Very low	No idea
1	I constantly communicate with my close people	5	4	2	1	3
2	If I need money, my close people lend me	5	4	2	1	3
3	There is a special person in my life that I share my most private feelings with her or him	5	4	2	1	3

Continued on next page

	Items	Very high	High	Low	Very low	No idea
4	My close people want me to be in their gatherings	5	4	2	1	3
5	To cope with my sorrows and troubles, I resort to religious practices (Pray, religious rituals, going to places of pilgrimage)	5	4	2	1	3
6	I feel that my close people love me	5	4	2	1	3
7	I feel that my presence is useful to my close people	5	4	2	1	3
8	When my close people approve of my work, I feel I have succeeded in life	5	4	2	1	3
9	My close people put me under too much expectations	1	2	4	5	3
10	My close people listen patiently to me	5	4	2	1	3
11	Whenever I feel upset, my close people sympathize with me	5	4	2	1	3
12	My close people express affection to me (cuddling, hugging, loving words)	5	4	2	1	3
13	I participate in charities (charity, volunteerism)	5	4	2	1	3
14	When my close people pray for me, I feel good	5	4	2	1	3
15	I feel that my close people are limiting my powers in life	1	2	4	5	3
16	I feel that I am important for my close people	5	4	2	1	3
17	My close people are always ready to help me	5	4	2	1	3
18	I hope to life	5	4	2	1	3
19	My close people appreciate my attention and help	5	4	2	1	3
20	I appreciate the attention and help of my close people	5	4	2	1	3
21	My close people pay attention to what's happening to me	5	4	2	1	3
22	My close people can always count on my help	5	4	2	1	3
23	My close people respect me	5	4	2	1	3
24	When I get sick, my close people take care of me (taking a doctor's appointment, going to the hospital, long-term care)	5	4	2	1	3
25	I go out with my close people for fun and leisure time (Cinema, theater, park, restaurant, religious ceremony, swimming pool, travel, party)	5	4	2	1	3
26	My close people trust me	5	4	2	1	3
27	I have a close and intimate relationship with my close people	5	4	2	1	3
28	If needed, my close people help me with my daily routine (buying household goods, housework, repairing equipment, commuting)	5	4	2	1	3
29	I enjoy spending time with my close people (family, friends)	5	4	2	1	3
30	I feel god is always with me	5	4	2	1	3
31	My close people support my actions	5	4	2	1	3
32	I feel lonely	1	2	4	5	3
33	I encourage my close people to pursue their interests	5	4	2	1	3
34	I’m satisfied with my relationships with my close people	5	4	2	1	3

AIMS Press

© 2020 the Author(s), licensee AIMS Press. This is an open access article distributed under the terms of the Creative Commons Attribution License (http://creativecommons.org/licenses/by/4.0)
